# Salivary Gland Choristoma: A Rare Finding at the Gastroesophageal Junction

**DOI:** 10.7759/cureus.7138

**Published:** 2020-02-28

**Authors:** Rachel Hanke, Isin Comba, Richard Henriquez, Maria W. Crespo, Lakhinder Bhatia

**Affiliations:** 1 Internal Medicine, University of Central Florida College of Medicine, Orlando, USA; 2 Internal Medicine, University of Central Florida/HCA Healthcare GME, Orlando, USA; 3 Pathology, Osceola Regional Medical Center, Kissimmee, USA

**Keywords:** heterotopic salivary gland, choristoma, gastroesophageal junction

## Abstract

A choristoma is a tumor-like outgrowth consisting of heterotopic, histologically mature tissue located at an anatomically unusual part of the body. Salivary gland choristoma at the gastrointestinal junction (GEJ) is an extremely rare entity with only one other case reported in the literature. In this report, we present the case of an 87-year-old female with long-standing gastroesophageal reflux disease (GERD) history who was incidentally found to have salivary gland choristoma at GEJ through an upper endoscopy-guided biopsy. We suggest that the finding of salivary gland choristoma at the GEJ could be metaplasia secondary to the patient’s long-standing history of GERD with esophagitis.

## Introduction

Heterotopic salivary gland tissue (HSGT) has been most commonly described in the head and neck region including the middle ear, gingiva, hard and soft palate, and external auditory canal. Malignancies arising from HSGT of the head and neck region have also been reported in the literature [1,2]. The gastrointestinal tract is an unusual site of HSGT with only a few cases reported in the literature at the following anatomic locations: lower esophagus, sigmoid colon, and rectum [3-5]. In this case report, we present a case of salivary gland choristoma at the gastrointestinal junction (GEJ), an extremely rare entity with only one other case reported in the literature [6]. Our case was diagnosed incidentally in an 87-year-old female through an upper endoscopy-guided biopsy of the nodule to exclude malignancy.

## Case presentation

An 87-year-old female presented with acute-onset left lower leg pain and swelling. The patient’s past medical history was significant for gastroesophageal reflux disease GERD (she was on daily famotidine), hypertension, and chronic kidney disease. On initial evaluation, vital signs were within normal limits and stable. Her laboratory tests were remarkable for elevated serum creatinine at 1.66 mg/dl, reduced estimated glomerular filtration rate at 29 ml/min, and microcytic anemia with initial hemoglobin at 7.9 gm/dL and mean corpuscular volume at 75.3 fL. Further investigation revealed normal coagulation panel. The iron panel was consistent with iron deficiency anemia. Doppler ultrasound showed an acute non-occlusive deep vein thrombosis in the left common femoral and femoral and popliteal veins. She was started tentatively on a therapeutic dose of enoxaparin.

**Figure 1:**
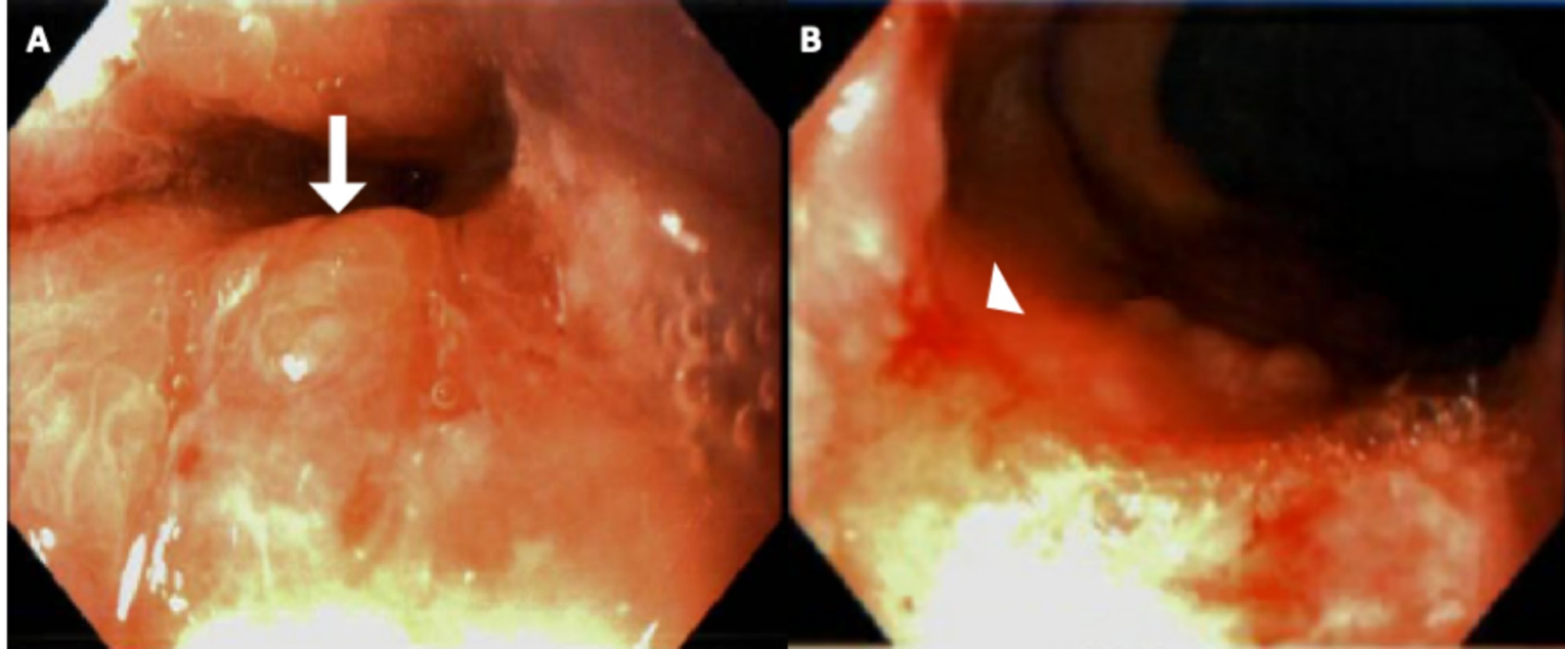
EGD findings EGD showed a small nodule at the GEJ (A, arrow) and reflux esophagitis (B, arrowhead) in the lower one-third of the esophagus EGD: Esophagogastroduodenoscopy; GEJ: gastrointestinal junction

**Figure 2:**
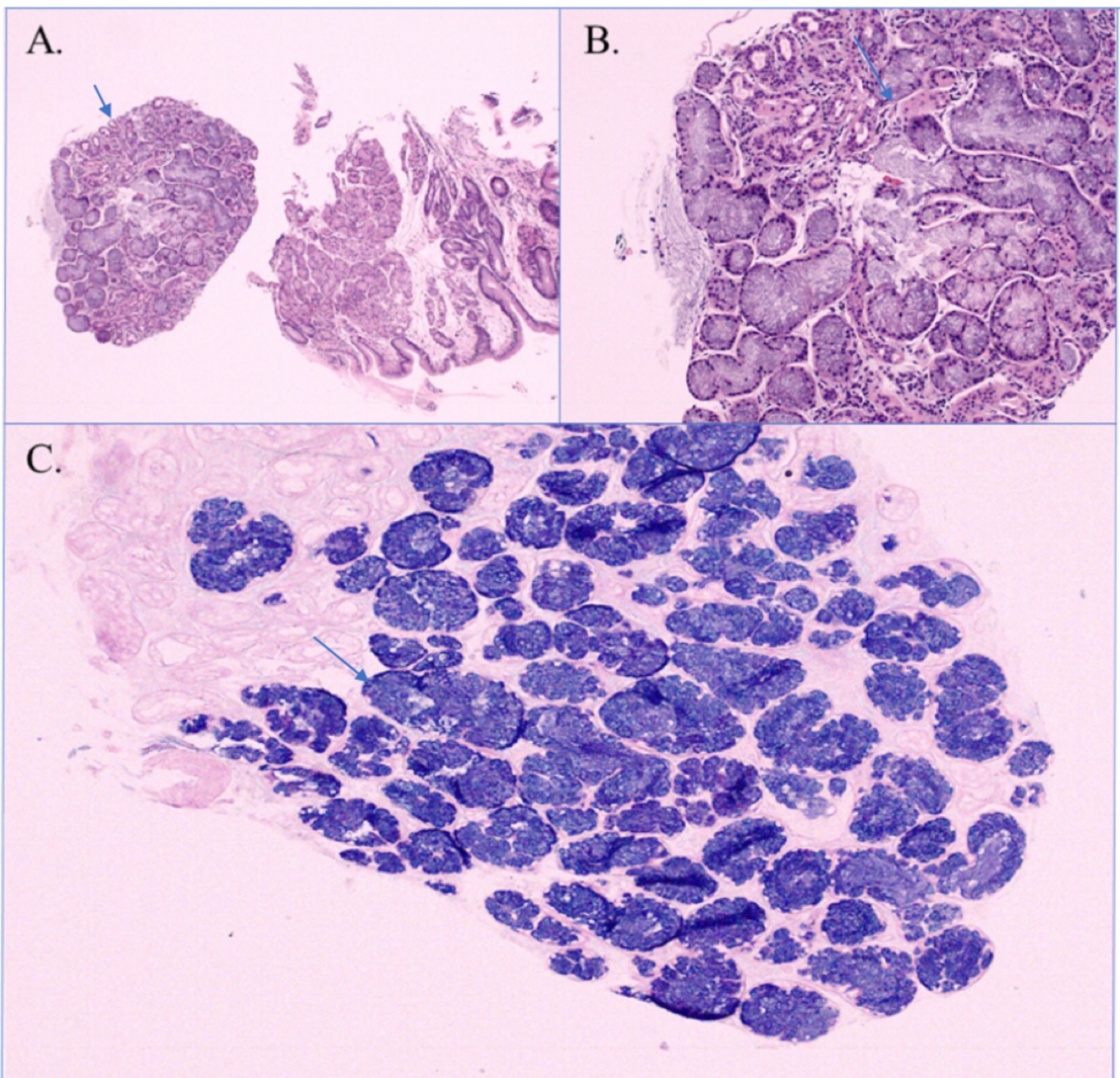
Biopsy findings On microscopic exam of the esophageal nodule, a focal glandular tissue was noted next to the esophagogastric junction-type mucosa with mild to moderate chronic inflammation (A). The focal glandular tissue was consistent with heterotopic salivary gland tissue (B, C)

During the hospitalization, the patient underwent a gastrointestinal evaluation for acute on chronic anemia due to suspected gastrointestinal bleeding. Upper endoscopy showed a large hiatal hernia, reflux esophagitis mainly in the lower one-third of the esophagus, and a small nodularity at the GEJ (Figure 1). Biopsy of the nodule demonstrated a GEJ-type mucosa with mild-to-moderate chronic inflammation, mild acute inflammation, and focal glandular tissue consistent with heterotopic salivary gland tissue (Figure 2). No intestinal metaplasia or dysplasia was noted. 

## Discussion

The human salivary glands are composed of three major glands (the parotid, sublingual, and submandibular) and numerous minor glands that take part in food digestion, lubrication, and immune defense. It is suggested that the parotid gland originates from the oral ectoderm, while the submandibular, sublingual, and intraoral minor salivary glands derive from the foregut endoderm [1].

Salivary gland choristoma at the GEJ is an extremely rare finding. Karim et al. recently reported three cases of HSGT at the GEJ, and only one of them was in the form of choristoma. All three cases presented in their report had a history of GERD, with one of the HSGT cases demonstrating grade-3 esophagitis and the other HSGT case having Barret’s esophagus with high-grade dysplasia [6]. Similarly, our patient had a history of long-standing GERD with esophagitis.

Repeated exposure of the esophagus to gastric contents through reflux triggers protective mechanisms in the esophagus resembling salivary gland function. An increase in the rate of mucin release from salivary glands and esophageal mucosal and submucosal glands into the mucus-bicarbonate layer is seen with decreasing pH associated with GERD [7]. Exposure to gastric contents has also been found to increase the secretion of prostaglandin E2 from salivary glands [8].

It is well known in the literature that intestinal columnar metaplasia at the GEJ can occur secondary to chronic acid exposure and inflammation in the setting of GERD. The origin of these intestinal glandular cells remains unknown. The presence of multipotent stem cells or the intrinsic ability of native squamous epithelium, submucosal glands, squamocolumnar junction cells or circulating bone marrow stem cells to dedifferentiate are proposed mechanisms [9]. The latter has been supported by the fact that the embryonic esophagus is lined by columnar epithelium [9,10]. Furthermore, pancreatic acinar metaplasia at the GEJ is a relatively common finding, but the causative relationship between GERD and pancreatic acinar metaplasia is obscure [11]. Likewise, our patient presented with a finding of unclear origins, demonstrating the relevance of keeping salivary metaplasia in the differential and the importance of further investigations.

In this case report, we aimed to enhance the understanding of clinicians about the pathophysiology of glandular metaplasia in response to chronic inflammation at the GEJ. Development in these biological mechanisms could also contribute to studies investigating cell-based therapy for salivary gland regeneration post-radiation therapy and in patients with Sjogren’s syndrome [1].

This work has been already presented as an abstract (https://insights.ovid.com/crossref?an=00000434-201910001-01841).

## Conclusions

We reported an extremely rare case of salivary gland choristoma at the GEJ. Based on our literature review, this finding could be a metaplastic change in the setting of chronic inflammation as a result of reflux esophagitis. However, the biological and clinical significance of the salivary gland choristoma at the GEJ is yet to be investigated. Further studies are warranted to examine the association between these two.
